# Transplacental transfer of anti-malarial antibodies: a systematic review

**DOI:** 10.3389/fimmu.2026.1821434

**Published:** 2026-06-19

**Authors:** Abebe Muche Belete, Abdouramane Camara, Taklo Simeneh Yazie, Lucas N. Amenga-Etego, Yaw Aniweh

**Affiliations:** 1 West African Center for Cell Biology of Infectious Pathogens (WACCBIP), College of Basic and Applied Science, University of Ghana, Accra, Ghana; 2 Crick Africa Network , The Francis Crick Institute (Crick), United Kingdom; 3 Department of Pharmacy, College of Health Science, Debre Tabor University, Debre Tabor, Ethiopia

**Keywords:** IgG, malaria, maternal-foetal immunity, newborn, transplacental transfer

## Abstract

**Background:**

Pregnant women and young children bear the highest burden of malaria morbidity/mortality. While transplacental transfer of malaria antibodies protects the child, the efficiency varies by antigen and maternal factors. This systematic review assessed transplacental transfer of malaria antibodies, factors associated with reduced transfer and identified knowledge gaps for future research.

**Method:**

We searched PubMed, Scopus, Web of Science and HINARI from the inception of databases until January 2025. We included published articles reporting anti-malaria antibodies in the mother and newborn, placental transfer and factors associated with reduced transplacental transfer of antibodies against malaria antigen. Two independent reviewers conducted the selection of articles, data extraction and risk of bias assessment. Meta-analyses of malaria prevalence were performed on the peripheral blood of mothers, on the placenta, and on the cord blood of newborns.

**Result:**

Forty-two studies (n=8687 pregnant women and newborns) were included in the analysis. Twelve studies reported the prevalence of malaria among mothers detected peripherally, 13 for placental malaria, and five studies for newborns. The pooled prevalence of peripheral and placental malaria was found to be 31.27% (95%CI: 24.9, 38.45), and 30.65% (95%CI: 20.29, 43.43), respectively. The pooled prevalence of malaria in the newborn was 9.53% (95%CI: 4.18, 20.29). IgG1 and IgG3 against malaria antigen were the predominant IgG that placentally transferred efficiently. IgG against circumsporozoite protein was more transferred to the newborn than those against the merozoite surface protein 3. Factors such as HIV infection, placental malaria, hypergammaglobulinemia in mothers, low birth weight, and primigravida were associated with reduced maternal transfer of antibodies against malaria antigen to newborns.

**Conclusion:**

Maternal transfer of antibodies against malaria antigen to newborns was IgG subclass and antigen-dependent. Vaccination strategies promoting high potential of transplacental transfer in pregnant women may enhance child protection against severe malaria.

**Systematic Review Registration:**

https://www.crd.york.ac.uk/PROSPERO/view/CRD42024609217, identifier CRD42024609217.

## Background

Pregnant women and children under 5 are particularly susceptible to infection, including malaria (1). Maternal antibodies cross the placenta via the FcRn receptor in placental syncytiotrophoblasts (2), providing some protection against infectious pathogens to the newborn. Therefore, an alternative strategy is maternal vaccination (2). This approach had successfully protected newborns against tetanus (3) and pertussis (4), and vaccines that were primarily offered to protect the mother during pregnancy, such as influenza (5, 6) and COVID-19 vaccines (7).

The two malaria vaccines R21 and RTS, S have been shown to be safe and effective in preventing malaria in children (8, 9) and are expected to have a high public health impact. No malaria vaccine has yet been approved for pregnant women. However, the *P. falciparum* sporozoites (PfSPZ) vaccine has shown promise. In phase-2 clinical trials, it demonstrated safety and significant efficacy against *P. falciparum* parasitemia and clinical malaria in women of childbearing age, with protection extending to subsequent pregnancies (10).

Transplacental transfer of immunoglobulin G begins as early as 6 weeks of gestation and increases slowly until the 28th week and peaks at term (11, 12). Maternally derived antibody protects the child from severe malaria. A longitudinal study showed that maternally-derived, cord-blood anti–PfSEA-1 antibodies predict decreased risk of severe malaria in infants. Also, vaccination of mice with PbSEA-1 before pregnancy protects their offspring from lethal *P. berghei* challenge (13). However, other findings showed that increased malaria infection occurred at the age of 9–12 months. Different factors may play a role in increasing malaria risk, such as breastfeeding and foetal haemoglobin level (14). High levels of IgG to erythrocyte binding antigen (EBA140 and EBA175, IgG1 to EBA175) and merozoite surface protein 142 (MSP142), and IgG3 to EBA140 and MSP5 were independent predictors of protection from clinical malaria during the first year of life. By contrast, high levels of IgG, IgG1, and IgG2 to the Variable 2 Chondroitin Sulphate A (VAR2CSA) Duffy Binding like domain (DBL)1–2 and IgG4 to DBL3–4 were significantly associated with an increased risk of clinical malaria (15).

The level and transplacental transfer of IgG vary with antigen, strain-specific and geographic setting. Antibodies to MSP2 3D7 were higher in mothers compared with the umbilical cord (16). However, IgG to MSP2 FC27 was the inverse (17). The transfer efficiency for total IgG against CSP was higher compared to MSP 3 (18, 19).

Several factors have been associated with reduced transplacental transfer of antibodies against malaria antigen, such as; placenta integrity (20), maternal HIV and malaria infection, maternal antibody level, gestational age, antigen specificity, malaria transmission intensities (endemicity), avidity of the antibodies (19), IgG isotypes, gravida status, and low birth weight. Placental transfer of malaria-specific antibodies, including total IgG and IgG1, have been shown to be reduced in women living with HIV (16). Antibody transfer increases with gestational age (12, 19). At delivery, IgG levels in newborns are equal to or exceed maternal IgG levels. However, this phenomenon is antigen-dependent. For instance, the levels and transfer ratios were higher for IgG against apical membrane antigen 1 (AMA1), EBA-175, and MSP1as compared to IgG levels against Liver Stage Antigen 1 (LSA1), circumsporozoite protein (CSP) and Ring-infected Erythrocyte Surface Antigen (RESA) (12).

Much as this area of research is crucial in the development and deployment of interventions, there has not been a systematic review of the transplacental transfer of antibodies against malaria antigen. Hence, to address this gap, we systematically synthesized evidence on the transplacental transfer of these antibodies across IgG subclasses and associated factors. We also conducted a meta-analysis on the prevalence of malaria infection in mothers and, in the placenta, and among newborns. This finding provides insights into the immunity of transplacental transfer of antibodies against *Plasmodium falciparum* antigens from mothers to newborns.

## Materials and methods

### Protocol and registration

We followed the guidelines and instructions in the updated protocol of Preferred Reporting Items for Systematic Review and Meta-analysis (PRISMA) (21) (**Supplementary 1 Table**). The protocol was prospectively registered with PROSPERO (registration number: CRD42024609217). Research question: Which malaria-specific antibody is transferred most efficiently to newborns across the placenta? What factors are associated with the transplacental transfer of malaria-specific antibodies anti-malarial antibodies?

### Eligibility criteria

Following the CoCoPop framework, the eligibility of studies was determined as follows:

### Inclusion

Population: We included pregnant women.

Condition: We considered studies that report comparing maternal and newborn IgG to malaria antigen during antenatal care or delivery. Mainly focused on IgG and its subclasses. Additionally, studies reporting on the placental transfer of antibodies against malaria antigens were included. The placental transfer of antibodies against malaria antigens was reported as the ratio of newborn antibodies against malaria antigen to mother antibodies against malaria antigens. If the ratio was ≥1 show efficient transfer of antibodies against malaria antigens to the newborn.

Context: We included studies conducted in the community or hospital that reported IgG against malaria antigen on either the mother’s or newborn’s side or both. We considered studies conducted around the globe and published in English.

Types of study: We included observational and randomized controlled trial studies.

### Exclusion

We excluded studies if they did not report the outcome of interest, i.e., transfer of IgG against malaria antigen, and we excluded those with unclear or insufficient data on IgG transfer measurements. We excluded conference or proceedings, non-peer-reviewed or grey literature, retracted articles, studies on animals, case series, case reports, letters, commentaries, and editorials.

### Information source

We used the Peer Review of Electronic Search Strategies (PRESS) methodology for systematic reviews in our search strategies (22). Each reviewer reviewed the search strategies. We searched articles from four electronic databases (i.e., PubMed, Scopus, Web of Science, and Research4life). The search strategy was designed to include studies published from inception of the databases to 25 October 2025.

### Search strategies

We used the following Medical Subject Heading (MeSH) terms and free text phrases in the search: ‘pregnant women’, ‘foetus’, ‘foetal’, ‘maternofoetal’, ‘transplacental antibody transfer’, ‘transfer efficiency’, ‘maternofoetal transfer’, ‘IgG’, ‘Immunoglobulin’, ‘IgG1’, ‘IgG2’, ‘IgG3’, ‘IgG4’, ‘total immunoglobulin’, ‘IgA’, ‘malaria’, ‘*Plasmodium falciparum*’, ‘*Plasmodium vivax*’, ‘*Plasmodium malariae*’, ‘*Plasmodium ovale*’. We used Boolean operators for combining the search terms. The specific search strategies for each database are included in (**Supplementary Material 2****).**

### Selection of studies

Records retrieved from each database were exported to EndNote version 8. After duplicates were removed, two review authors (AMB and TSY) performed the screening process. A two-step screening process was conducted initially, including title and abstract screening to identify potentially relevant studies, followed by complete text evaluation of those studies that passed the title and abstract screening against predetermined eligibility criteria were recorded, while we recorded the reason for exclusion of ineligible studies. Any disagreement between the authors was solved by discussion, and if needed, the third author was invited. The reviewers presented substantial agreement (Cohn’s kappa=0.8) throughout the screening process.

### Quality assessment

The same authors (AMB and TSY) assessed the quality of the included studies using the Newcastle-Ottawa Scale tool (23). The tool consists of representativeness, response rate, method of comparability of the subjects, and appropriateness of the statistical analysis. This item is out of 10, and then studies scored five and above were included (**Supplementary Material 3****).**

### Data extraction

We developed a data extraction format using Excel based on the Joanna Briggs Institute (JBI) data extraction form (24). The following data were extracted from the included studies: Author name, year of publication, country, the type of *Plasmodium* species, anti-malarial antibody, study date, sample size included in maternal and cord, prevalence of maternal malaria, prevalence of placental malaria, maternal IgG, newborn IgG, placental transfer ratio and associated factors.

### Assessment of risk of bias

Two authors (AMB and TSY) assessed the risk of bias of included studies using the Hoy risk of bias assessment tool (25). The tool is out of ten: 8–10 is low risk of bias, 5–7 is moderate, and ≤4 is high risk.

### Data synthesis and statistical analysis

The primary outcome of this systematic review was transplacental transfer of antibodies against malaria antigen. To address this outcome, we performed a narrative synthesis, stratifying findings by antigens: pre-erythrocytic, erythrocytic stage, and placental antibodies. A quantitative pooled meta-analysis of transplacental transfer ratio was not conducted for the following reasons: the included studies were heterogeneity in terms of the diversity of antigen reported, method antibody measurement/unit. On the other hand, data were available from the included studies to conduct quantitative data pooling on the prevalence of malaria in peripheral, placental and cord. This pooling (i.e. prevalence/proportionate meta-analysis) using the inverse variance heterogeneity model (26) and the double arcsine transformations to stabilize the variance (27) was conducted. The secondary outcome of this review was the identification of factors associated with the transplacental transfer of antibodies against malaria antigens. Quantitative pooling of factor data was not performed, as the included studies employed heterogeneous analytical approaches to measure and report associations, including odds ratios, beta coefficients, and qualitative directional descriptions of increased or decreased transfer. Therefore,a narrative data synthesis was conducted to systematically summarize and contextualize the factors associated with reduced transplacental transfer of antibodies against malaria antigens across the included studies. All data were analysed using R studio version 4.4.3.

## Results

### Description of studies

**Figure 1:**
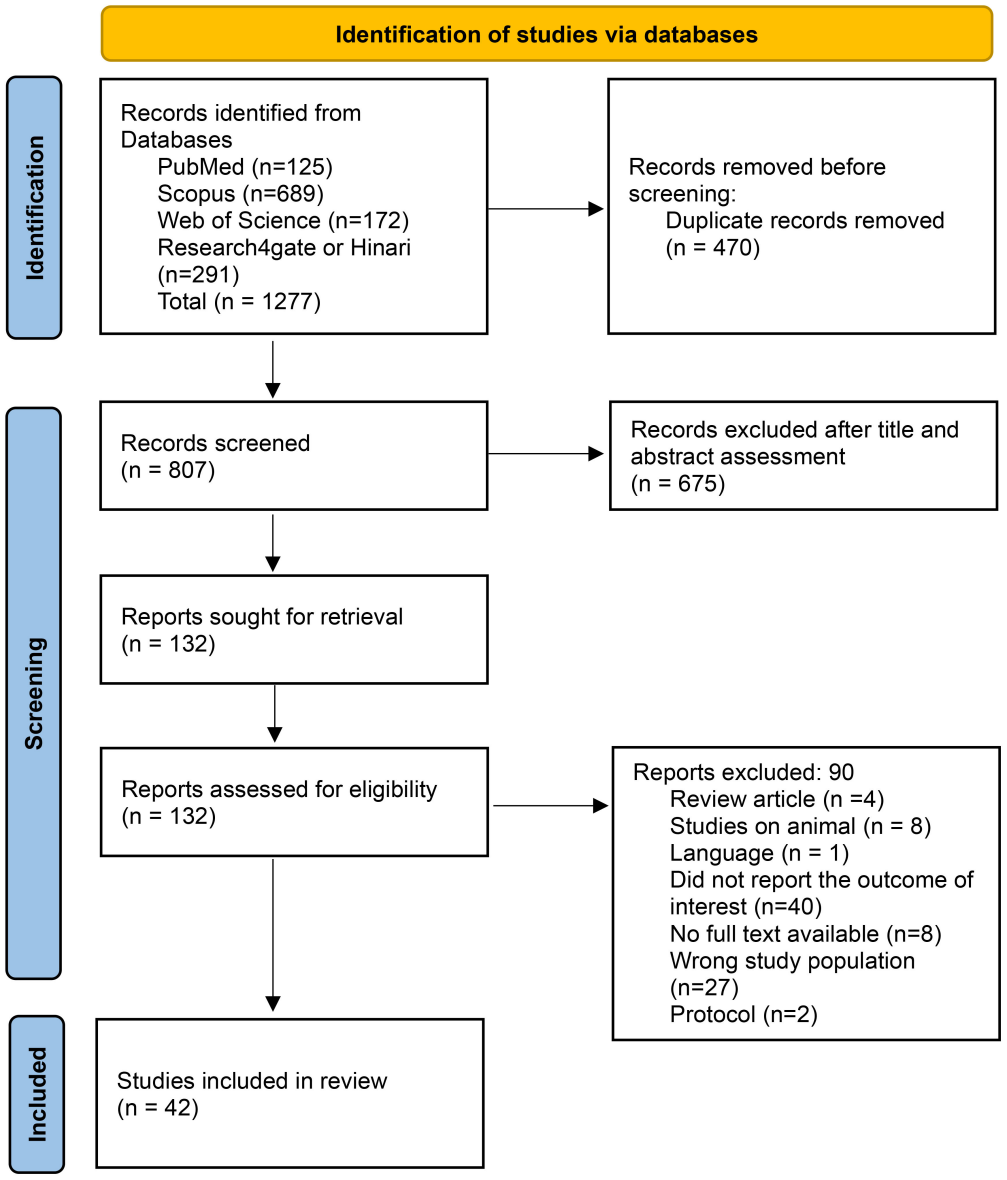
PRISMA flow chart shows the included studies.

We identified a total of 1277 records from a database search. After 470 duplicated articles were removed, 807 articles were screened for title and abstract. We reviewed 132 articles for further details and excluded 90 articles. We included 42 studies in the final review (**Figure 1**).

### Characteristics of included studies

**Figure 2:**
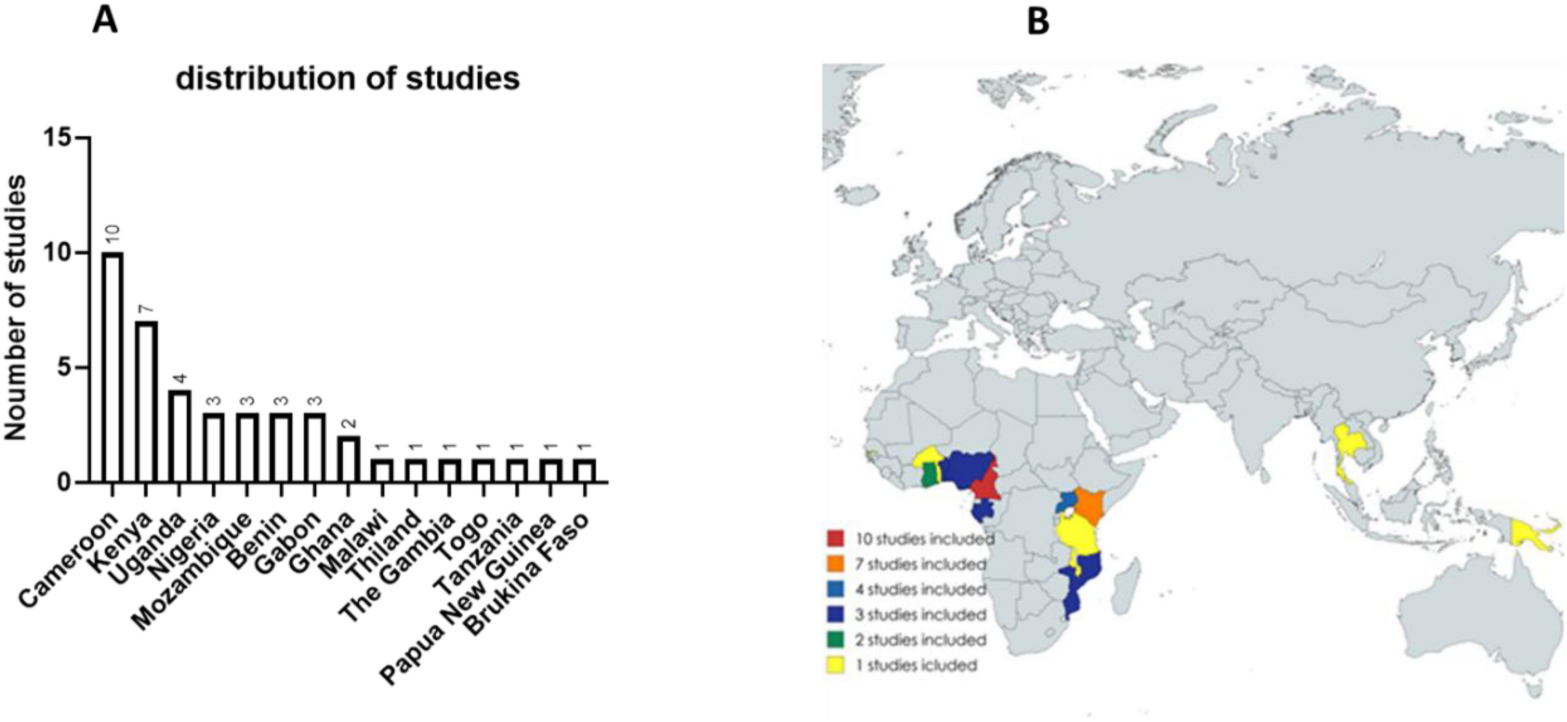
Distribution and geographic representation of included studies. **(A)** Distribution of studies by country. **(B)** A world map illustrating the geographic distribution of included studies. Countries are color-coded according to the number of studies included.

Forty-two studies were included in the review. The study, country, plasmodium species, sample size, antimalaria antibody measurement, placental malaria, and maternal malaria are presented in **Table 1**. We identified ten (23.8%) studies from Cameroon (12, 19, 28, 30, 31, 33, 41, 43, 48, 58), seven (16.6%) from Kenya (32, 35, 45, 50, 54, 56, 61), four (9.5%) from Uganda (14, 49, 55, 60), three (7.1%) from each Nigeria (29, 37, 44), Mozambique (51, 53, 62), Benin (17, 40), and Gabon (36, 39, 59), two (4.7%) from Ghana (18, 57), and one (2.3%) each from Malawi (34), Thailand (38), The Gambia (42), Togo (46), Tanzania (13), Papua New Guinea (52), and Burkina Faso (15) (**Table 1**). **Figure 2** displays the distribution of each article.

**Table 1:** Characteristics of included studies.

Author year		Country		Location	Malaria transmission	Study period		Pl.sp	Antibodies measured	SSP	SSM	SSC	Method for dx	Method for IgG
Achidi, Eric A. et al., 2005 (28)		Cameroon		Fako,South West	Perennial	1999-2001		Pf	Pf schizont stage	627	444	407	Microscope	ELISA
Achidi EA. et al., 1995 (29)		Nigeria		IgboOra	Perennial	February-May 1991		Pf	PF155, (EENV)6, CSP	117	63	51	Microscopic	ELISA
Akum AE. et al., 2005 (30)		Cameroon		Fako division, South West	NR	MayA-August 2000		Pf	Blood stage	110	110	110	microscope	ELISA
Alonso S. et al., 2021 (16)		Mozambique		Manhica district, Southern mozambique	NR	2011-2012		Pf	DBLD-3&4, EBA 140, EXP1, MSP 1, 2, 5, RH4.2	NR	332	303	qPCR	Quantitative suspension array
Anchang-Kimbi JK et al., 2016 (31)		Cameroon		South West	Perennial	March-October 2007		Pf	Crude plasmodium falciparum	NR	271	NR	microscope	ELISA
Ayisi, J. G., et al., 2003 (32)		Kenya		Kisumu	NR	April-October 1997		Pf	MSP-1, MSP-2, MSP-3, RAP1, NANP	NR	1087	NR	Microscope	ELISA
Babakhanyan, A.2016 (33)		Cameroon		Yaoundé	NR	2014-2015		Pf	CSP, AMA-1, MSP 1	107			microscope	Multianalyte platform assay
Boudová S., et al., 2017 (34)		Malawi		Blantyre	endemic	NR		Pf	AMA-1, MSP-1, RH5	33	33	33	qPCR	Protein microarray
Branch et al., 2000 (35)		Kenya		Western Kenya Lake Victoria	Throughout the year	1992-1993		Pf	MSP1		33		Microscope	ELISA
Brustoski K et al., 2005 (36)		Gabon		Lambare´ne´	perennial	May-December 2003		Pf	DBL25, DBL78, DBL120, DBL132	85			microscopic	ELISA
Onyenekwe. et al., 2004 (37)		Nigeria		NR	NR	NR			P. falciparum hrp-2 antigen		21	21	Microscope	ELISA
Charnaud SC et al., 2016 (38)		Thailand		north-west Thailand	Low transmission	1998-2000		Pf&PV	PfAMA1, PfEBA175, PfEBA140RII, PfEBA140RIII-V, PfMSP2, PfRh2		37		Microscopic	ELISA
Chizzolin C., et al., 1991 (39)		Gabon		Haut-OgoueeProvinc	perennial	1994-1995		Pf	Pf asexual stage	59			Microscopic	Immunofluorescent assay
Dechavanne C, et al., 2015 (17)		Benin		Tori Bossito district(meso-endemic)	Meso-endemic	2007-2010		Pf	AMA1, MSP1, MSP2, MSP2, MSP3, GLURP-R0, GLURP-R2	531			RDT or microscopic	ELISA
Dechavanne C, et al., 2017 (40)		Benin		Tori-Bossito	Meso-endemic	2007-2008		Pf	MSP1-19, MSP3, MSP2, GLURP-R0, GLURP-R2	497			Microscopic	ELISA
Deloron P, et al., 1997 (41)		Cameroon		Ebolowa	hyperendemic with perennial transmission.	1992-1993		Pf	Anti-Plasmodium falciparum	150			Microscope	ELISA
Duah NO, et al., 2010 (42)		The Gambia		Greater Banjul Area	Highly seasonal	2003-2004		Pf	AMA1, MSP1-19, MSP2, MSP3			53	NA	ELISA
Fodjo, B.A.Y., et al., 2016 (43)		Cameroon		Ngali II and Ntoues-song, yaounde,	High in Ngali II and Ntoues-song Low in Yaounde	2001-2003		Pf	VAR2CSA DBL			21	Microscopic	ELISA
Ibeziako, P. A., & Williams, A. I. O. 1980 (44)		Nigeria		Ibadan	NR	NR		Pf	Malaria antigen (no specific antigen)	60	60		Microscopic	Immunodiffusion technique
Kayatani AKK., et al., 2023 (19)		Cameroon		Yaoundé	~13 infectious bites per person-year	1996-2001		Pf	AMA-1, EBA-175, MSP1-142, MSP2, MSP3, DBL5,	80	80	80	Microscopic	Luminex-based multiplex assays
Kayatani, A.K.K., et al., 2022 (12)		Cameroon		Yaoundé	~13 infectious bites per person-year	1996-2001		Pf	AMA-1, EBA-175, MSP1-42, MSP2, MSP3, DBL5-VAR2CSA, CSP, LSA1	320	320	320	microscopic	Luminex-based multiplex assays
King CL, et al., 2002 (45)		Kenya		Kwale District	Holoendemic	NR		Pf	MSP-1 (19)	92		92	microscopic	ELISA
Kirch AK, et al., 2004 (46)		Togo		Central Togo	NR	NR		Pf	Pf antigens	NA			NA	ELISA
Kurtis JD., et al., 2019 (13)		Tanzania		Muheza Designated District	P. falciparum–holoendemic	NR		Pf	MSP)-1–19, MSP-3, MSP-7, LSA-N (28–150 aa), LSA-C (RAMA)-E, and RAMA-Pr	647			NR	multiplexed, bead-based assay
Author year	Country	Location		Malaria transmission		Study period	Pl.sp	Ab measured	SSP	SSM	SSC	Malaria dx	Method of IgG profile
Leonard et al., 2023 (47)	Nigeria	Nation wide		Including high transmission		July-December 2018	Pf,pv,pm,po	AMA-1, MSP1, CSP, LSA1, GLURP-R0,	41			NR	multiplex bead assay
Lissom A., et al., 2024 (48)	Cameroon	Yaounde		Holoendemic		2017-2018	Pf	QβMSP3, QβUB05 and QβUB05-MSP3		88		RDT and microscopic	ELISA
Luga Ajju, A., 2017 (49)	Uganda	Wakiso district		meso-endemic		2012-2013	Pf	Anti-pf	131		131	RDT and microscopic	ELISA
Malhotra I., et al., 2009 (50)	Kenya	Kwale District		Holoendemic area stable, with seasonal variation related to rainfall		NR	Pf	MSP142, AMA1, and EBA175-Region II	586			Microscopic	ELISA
Mayor, A., 2018 (51)	Mozambique	Manhiça		perennial and of moderate intensity with a warm rainy season		2005-2007	Pf	MSP1, EBA175, AMA1, DBLα	207			Microscopic & RTqPCR	ELISA
McLean et al., 2017 (52)	Papua New Guinea	Thailand-Myanmar Border Area		NR		2005-2007	Pf	PfEBA175RII, PfMSP2, PfAMA-1, PfDBL5	118			Microscopic	ELISA
Moro, L., et al., 2015 (53)	Mozambique	Manhiça district		Perennial		2002–2010	Pf	MSP1 19, EBA175, AMA1, & parasite lysate	187			Microscopic & qPCR	ELISA
Mortazavi SE, et al., 2023 (14)	Uganda	Kasangati		Moderate transmission			Pf	Total Pf IgG		131	129	RDT & Microscopic	ELISA
Murungi LM, et al., 2017 (54)	Kenya	Kilifi County		two seasonal peaks in malaria transmission (May to August and October to November)		2001-2008	Pf	schizont lysate, AMA1(3D7), MSP-2, MSP-3, MSP-119 and PfRh2			130	Microscopic	ELISA
Natama HM, et al., 2023 (15)	Burkina Faso	Nanoro		seasonal and hyperendemic		NR	Pf	CSP-fl, CSP-Ct, CSP-NANP; AMA1, MSP142, MSP2, MSP3, MSP5, EBA140, EBA175, GARP, Rh5, DBL1-2, DBL3-4, PfEMP1	661			Microscopic & PCR	Quantitative suspension array technology
Okek, E. J., et al., 2023 (55)	Uganda	Busia District		NR		NR	Pf	HSP40Ag1, ETRAMP5Ag1, SEA, AMA-1, EBA140RIII-V, EBA175RIII-V, EBA181RIII-V, RH4.2, RH5, GLURP RII, MSP1-19, MSP2Dd2, MSP2CH150/9, RH22030, CSP			640	NA	MagPix multiplex bead array assay
Partey, F.D., et al., 2024 (55)	Ghana	Accra		NR		March-May 2022	Pf	MSP3, CSP	199	199		NR	ELISA
Ray JE, et al., 2019 (56)	Kenya	Kisumu County		perennially high		2013 - 2015	Pf	MSP2, MSP9, RH5, CSP, EBA140, EBA175, EBA181, MSP3, MSP1, MSP6, MSP7, MSPDBL1, MSPDBL2, AMA1	99			PCR	ELISA
Stephens, J. K., et al., 2017 (57)	Ghana	Madina, Accra		NR		NR	Pf	GLURP	121	121		NR	ELISA
Tassi Yunga, S., et al., 2022 (58)	Cameroon	Ngali II		257 infectious mosquito bites annually		2001-2004	Pf	AMA-1, AMA-1 (FV0), EBA-175, MSP-1, MSP-1, MSP-2, MSP-2, MSP-3, RESA, LSA-1, CSP	70			Microscopic & PCR	Multiplex Luminex assay
Tena-Tomás, C., et al., 2007 (59)	Gabon	Libreville		NR		NR	Pf	GST-DBL-α C1, GST-DBL-α C2, GST-DBL-α C3	42			Microscopic	ELISA
Tijani MK, et al., 2024 (60)	Uganda	Kasangati		NR		NR	Pf	Anti-PS	131			NR	ELISA
Zhou, Z., et al., 2002 (61)	Kenya	western Kenya		Hyperendemic (2–10 infected bites per person per month)		NR	Pf	CSP, LSA-1, MSP-2 FC27, MSP-2 3D7		14	20	Microscopic	ELISA

Key: AMA-1, Apical Membrane Antigen 1, DBLD-1-2, 3- 4, Duffy-binding like domains 1–2 and 3-4, EBA 140, Erythrocyte-binding antigen 140, EBA140RIII-V, Erythrocyte Binding Antigen-140 Region III-V, EBA175RIII-V, Erythrocyte Binding Antigen-175 Region III-V, EBA181RIII-V, Erythrocyte Binding Antigen-181 Region III-V, EXP1, exported protein 1, MSP 1, 2, 3,5,7, merozoite surface proteins 1, 2, 3, 5, 7, MSP1-19, 19kDa fragment of MSP1 molecule, MSP2Dd2, Merozoite Surface Protein 2, Dd2 allele, MSP2CH150/9, Merozoite Surface Protein 2, CH150/9 allele, MSP142,C-terminal 42 kDa cleavage of merozoite surface protein 1; RH4.2, reticulocyte-binding-homologue-4.2., Rh5, reticulocyte-binding protein homolog-5, liver stage antigen (LSA)-N, LSA-C, RAMA-E, rhoptry associated membrane antigen.

HSP40Ag1,Heat Shock Protein 40, type II, ETRAMP5Ag1, Early Transcribed membrane protein 5, SEA, Schizont Egress Antigen, GLURP RII, Glutamate Rich Protein R2, RH22030, Reticulocyte Binding Protein Homologue 22030, CSP, Circumsporozoite Protein

CSP-fl, circumsporozoite full length protein, CSP-Ct, C-terminal end of CSP, CSP-NANP, NANP repeat central region of CSP, AMA1, EBA140, erythrocyte binding antigen, EBA175, GARP, glutamic acid rich protein, PfEMP1, erythrocyte membrane protein 1

NR, Not reported, NA, Not available, SSP, sample size paired, SSM, sample size maternal, SSC, sample size cord

Eight studies reported on antibodies against circumsporozoite protein (CSP) antigen (12, 18, 29, 33, 47, 56, 58, 61) and seventeen studies reported antibodies against the merozoite surface protein (MSP) (12, 16–19, 32–35, 38, 45, 47, 51, 53, 56, 58, 61). Out of these, fourteen studies reported on antibodies against MSP1 (12, 16, 17, 19, 32–35, 45, 47, 51, 53, 56, 58), nine studies reported on antibodies against MSP2 (12, 16, 17, 19, 32, 38, 52, 56, 58, 61), six studies reported on antibodies against MSP3 (12, 17, 19, 32, 56, 58), each one reported on antibodies against MSP6, MSP7, MSP9, MSPDBL1, and MSPDBL2. Nine studies reported on antibodies against apical membrane antigen (AMA-1) (17, 19, 33, 34, 51–53, 56, 58). Nine studies reported on erythrocyte binding antigen (EBA) (12, 16, 19, 38, 51–53, 56, 58).

### Prevalence of malaria

**Figure 3:**
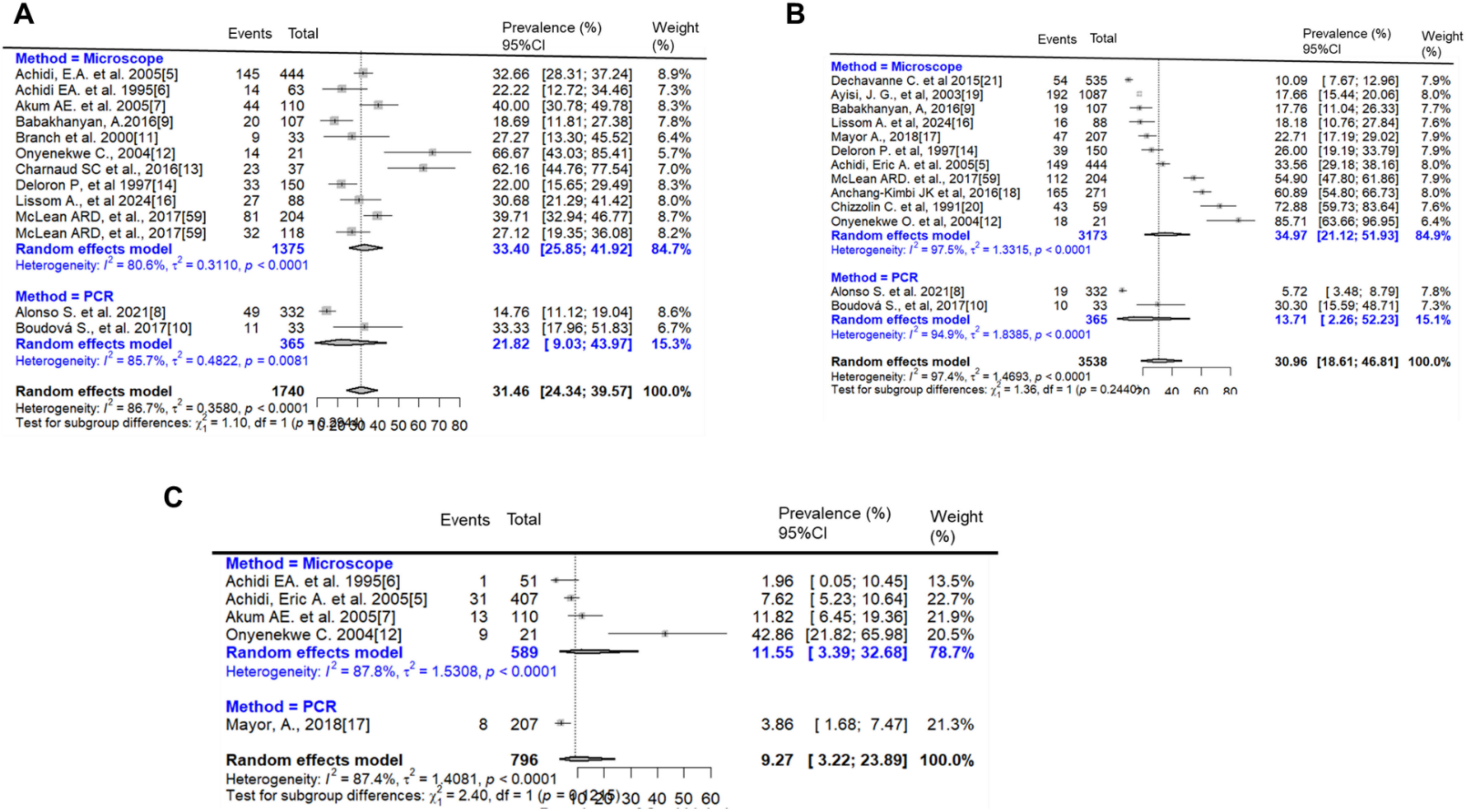
Forest plot showing the prevalence of malaria based on microscopic and PCR. **(A)** Pooled prevalence of peripheral malaria among women. **(B)** Pooled prevalence of placental malaria. **(C)** Pooled prevalence of malaria from cord blood of newborns. Diamonds represent pooled prevalence estimates with 95% CIs from random-effects models. Heterogeneity was assessed using the I² statistic and τ² (between-study variance). CI: confidence intervals, PCR: Polymerase Chain Reaction,.

To determine the pooled prevalence of malaria from included studies, the mother’s peripheral blood was tested at delivery using microscopic or/polymerase chain reaction (PCR) or both methods. Twelve studies examined the prevalence of peripheral malaria among mothers (16, 28–30, 33–35, 37, 38, 41, 48, 52). The pooled prevalence of peripheral malaria by microscope was 33.40% (95%CI:25.85, 41.92), I^2^ = 80.6% and that estimated by PCR was 21.82(95%CI: 9.03, 43.97), I^2^ = 85.7%. The overall pooled prevalence across both methods was 31.46% (95%CI: 24.37, 39.57), I^2^ = 86.7% (**Figure 3A**).

To determine the presence of malaria in the placenta, the placenta was examined at delivery. Thirteen studies reported the prevalence of placental malaria (16, 17, 28, 31–34, 37, 39, 41, 48, 51, 52), giving the pooled prevalence of 30.96% (95%CI: 18.61, 46.81), I^2^ = 97.4. when stratified based on diagnostic methods, the prevalence estimated by microscope was 34.97% (95%CI: 21.12, 51.93), I^2^ = 97.5%, and that estimated by PCR was 13.71% (95%CI: 2.26, 52.23), I^2^ = 94.9% (**Figure 3B**).

To determine whether the newborn has malaria, blood was taken from the umbilical cord and tested using either a microscope or PCR or both. Five studies evaluated the prevalence of malaria in newborns (28–30, 37, 51); with an overall pooled prevalence of 9.53% (95%CI: 4.18, 20.29), I^2^ = 87.4%. The prevalence estimated by microscope was 11.55% (95%CI: 3.39, 32.68) (**Figure 3C**).

### IgG profiles against malaria antigen in mothers and newborns

**Figure 4:**
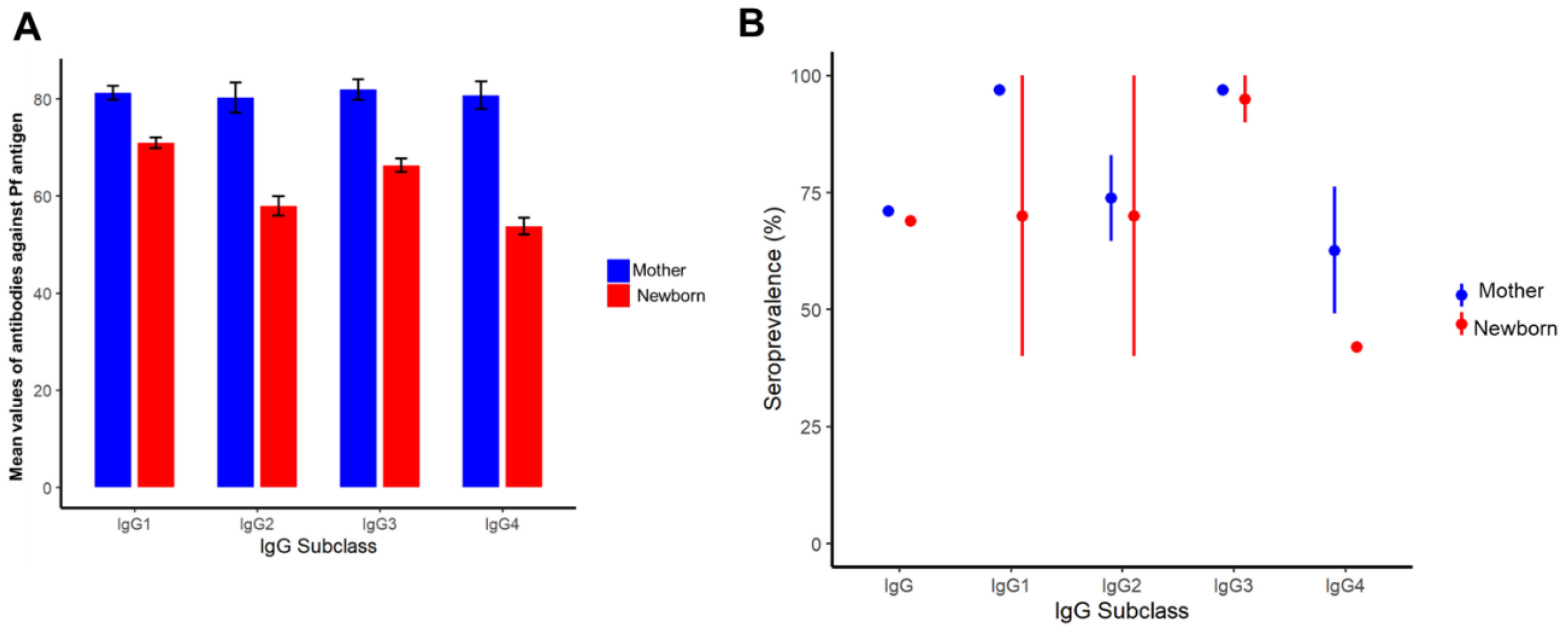
Overview of the mean and seroprevalence of *Plasmodium falciparum* IgG in both mother and newborn. **(A)** the mean value and standard deviation of IgG subclasses against *Plasmodium falciparum* antigen (41). The unit of measurements was Arbitrary unit. **(B)** the seroprevalence of *Plasmodium falciparum* against IgG and IgG subclasses. The seropositivity among mother (31, 41) and cord (39, 41). Because we used two different study so the vertical line indicates the range of the percentage reported from each study. Antibodies level higher in mother than newborn reflect findings reported within individual studies based on their own statistical analysis. No cross-study pooled statistical comparison was performed, as substantial heterogeneity in antigen targets, antibody measurement platforms, and reporting formats across the included studies. Blue colour is for mother and red is for newborn. IgG: immunoglobulin G.

I gG profiles against malaria antigens were conducted on blood collected from mother and newborn from the umbilical cord. The seroprevalence and mean levels of IgG and its subclasses against malaria antigen were higher in the mother compared to the newborn (13, 29–31, 39, 41, 44, 54, 55). From IgG subclass, IgG1 and IgG3 were higher than IgG2 and IgG4 in both mother and newborn (31, 41) (**Figures 4A, B**). Their transfer efficiency was reduced with placental malaria infection than without (17).

### Antibodies against circumsporozoite protein

The seroprevalence of IgG against PfCSP was 47.4% (38), with a higher geometric mean level in the mothers than in the newborns (61). HIV and placental malaria infections influence antibodies against CSP. IgG levels against CSP were lower in HIV+ mothers, and newborn IgG against CSP was significantly lower in neonates born to HIV+ mothers (33). Past and chronic placenta malaria were more frequently associated with a statistically significant increase of IgG and/or subclass levels against the C-termina, the central repeat region (NANP), as well as the full length of CSP (15). Among SARS-CoV-2 patients, the IgG and transfer efficiency levels against CSP antigen were higher than those against MSP3 antigen (18). However, Natama et al. reported higher levels of IgG and IgG subclass against MSP3 than CSP (15).

### Antibodies against apical membrane antigen

The seroprevalence of IgG against AMA-1 ranged from 66-93% in newborns (34, 54) and 64% in mothers (34). In both women with and without placental malaria, the mean IgG against AMA-1 was higher in the mothers than in the newborns (17). Additionally, IgG antibody level against AMA-1 in newborn was significantly lower than in the mother (33). IgG1 was higher than IgG3 against AMA-1 in newborns (42). The transplacental transfer was higher in women without placental malaria than with placental malaria (17).

### Antibodies against merozoite surface protein

**Figure 5:**
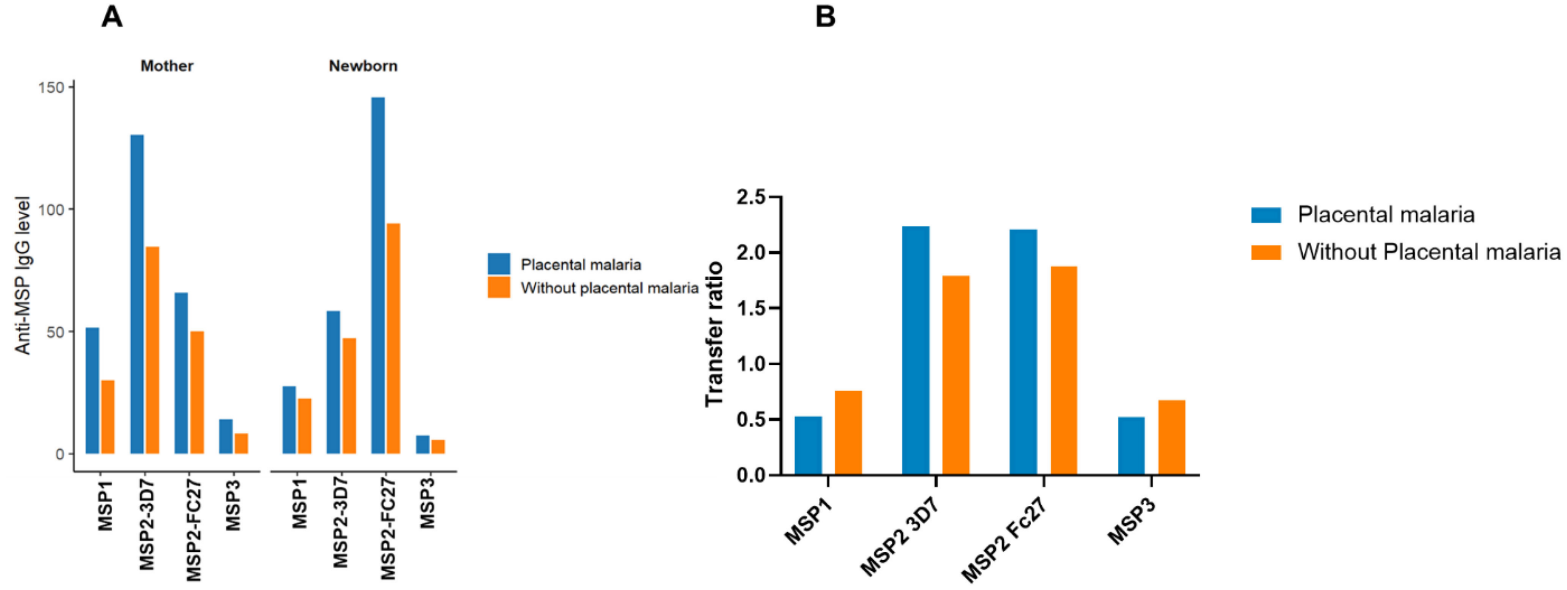
Antibodies against merozoite surface protein (MSP). **(A)** IgG levels against different MSP with placental malaria and without placental malaria both in mother and newborn. **(B)** the transfer ratio, which is newborn antibodies divided by mothers mean antibodies. Antibodies level higher in mother than newborn reflect findings reported within individual studies based on their own statistical analysis. No cross-study pooled statistical comparison was performed, as substantial heterogeneity in antigen targets, antibody measurement platforms, and reporting formats across the included studies. Blue colour is for mother and red is for newborn.

Fourteen studies reported on antibodies against MSP1 (12, 16, 17, 19, 32–35, 45, 47, 51, 53, 56, 58), nine studies reported on antibodies against MSP2 (12, 16, 17, 19, 32, 38, 52, 56, 58, 61), and six studies reported on antibodies against MSP3 (12, 17, 19, 32, 56, 58) (**Figure 5A**).

The seroprevalence of IgG against *Plasmodium falciparum* MSP was 68.4–93.0% among malaria-exposed women and 57–85.3% among the non-exposed group. The seroprevalence of IgG against *Plasmodium vivax* MSP was 57.9–61.4% in malaria-exposed women and 38.5–63.3% in malaria non-exposed women (38).

The seroprevalence of IgG against MSP1 among mothers was 62% (34) and varied among newborns (6% to 94%) (17, 35, 45, 54). The seroprevalence of IgG subclass against MSP1 was 62 - 73% for IgG1, 30% for IgG2, 26-70% for IgG3 (35, 42), and 33% for IgG4 (35). The geometric mean of IgG against MSP1 in those with and without placental malaria was higher in the mother than in the newborn (**Figures 5b, c**). The transfer without placental malaria was higher than that with placental malaria (17) (**Figure 5D**).

The seroprevalence of IgG antibody against MSP2-3D7 among mothers was 93% and 90% in newborns. The mean geometric value of IgG antibody against MSP2-3D7 was higher among placental malaria than without placental malaria, and the mother was higher compared to the newborn (**Figure 5B**). The placental transfer of IgG against MSP2 was higher in placental malaria than in the absence of placental malaria (17). IgGl antibody level against MSP1 b12 and MSP2 were low. IgG3 antibody level against MSP1 b12 and MSP2 were high (16).

The seroprevalence of IgG antibody against MSP2 Fc27 among mothers was 85.7%, and 75% among newborns. The mean geometric value of IgG antibody against MSP2 Fc27 was higher in placental malaria than without placental malaria (**Figure 5B**). The mean value of IgG antibody against MSP2 Fc27 among newborns in the placental malaria was higher than the mother’s IgG (**Figure 5C**). The placental transfer of antibodies against MSP2 Fc27 was higher among placental malaria than without placental malaria (17) (**Figure 5B**).

The seroprevalence of IgG1 antibody against MSP2-Dd2 and MSP2-ch150/9 among newborns were 28% and 25%, respectively. Seropositivity of IgG3 against MSP2-Dd2 among newborns was 85% and 83% for MSP2-ch150 / 9. The seroprevalence of IgG2 against MSP2-ch150 / 9 in newborns was 53% and 45% for IgG4 (42).

The mean geometric of IgG antibody against MSP3 was higher in placental malaria than without, and also higher in the mother than the newborn (**Figure 5A**). The transfer was lower in placental malaria than without (17)(**Figure 5B**). The seroprevalence of IgG1 antibody against MSP3-3D7 and MSP3-K1 among newborns were 66% each. The seroprevalence of IgG3 antibody against MSP3-K1 among newborns was 53% and 43% for seroprevalence against MSP3-3D7 (42). A case control study showed that the seroprevalence of total IgG antibody level against MSP-3(3D7) among sever malaria cases was 69% and 68% among those individuals without malaria (54).

### Antibodies against Duffy-binding-like domains

Six studies reported on antibodies against Duffy-binding-like domains (DBL) domain antigens of Plasmodium falciparum of erythrocyte membrane protein 1(PfEMP1), encompassing several domain variants including DBLα, DBL3–4 and GST-DBL-α. Among newborns, IgG reactivity to DBL peptide was 4-10% (36). The seroprevalence of total IgG against DBLα was 82.5% in women with *plasmodium* spp. parasitemia detected by light microscope at any time during pregnancy, compared with 78.9% in women without parasitemia during pregnancy (38). IgG1 antibody level against DBL3–4 were high levels, whereas IgG3 antibodies against DBL3–4 were low. IgG4 antibodies against DBL3–4 was the most efficiently transplacental transferred, followed by IgG3 and IgG2 (16). In a study of prenatal immune responses in Gabon, maternal IgG seropositivity to GST-DBL-α variants (C1, C2, and C3) ranged from 19% to 88%, while the corresponding cord blood seropositivity ranged from 41% to 83% (59).

### Antibodies against glutamate rich protein

The study revealed differences in the geometric mean antibody levels against GLURP-R0 and GLURP-R2 between pregnant women with and without placental malaria infection. Antibodies against GLURP-R0 IgG among women without placental malaria infection reported 5.49 μg/mL (95%CI: 0.63 to 10.36) in mother and newborn levels of 3.36 μg/mL (95%CI: -1.07 to7.80), with a log transformed placental transfer ratio of a geometric mean 0.61 (95%CI: -1.11 to 2.33). In contrast, women with placental malaria showed higher antibodies 7.41 μg/mL (95%CI: 2.31 to12.51) but comparable newborn levels 3.46 μg/mL (95%CI: -1.17 to 8.09) and a lower log transformed placental transfer ratio 0.47 (95%CI: -1.13 to 2.06). The original study reported negative lower confidence interval bounds for some cord blood IgG estimates. However, the methodology section did not clearly specify whether IgG concentrations were analysed or reported using log-transformed values.

The mean geometric levels against GLURP-R2 IgG among mothers with no placental malaria infection were 36.97 μg/mL (95%CI: 33.04 to 40.90), and 21.47 μg/mL (95%CI: 17.89 to 25.06) among newborns. The log transformed placental transfer ratio was a geometric mean of 0.57 (95%CI: -1.15 to2.29). Among mothers with placental malaria infection, the antibodies against GLURP-R2 among mothers were 54.62 μg/mL (95%CI: 49.45 to 59.78), and 22.70 μg/mL (95%CI: 18.73 to26.68) among newborns. The log transformed placental transfer ratio was a geometric mean of 0.42 (95%CI: -1.67 to2.51) (17).

In a low malaria transmission setting in a co-endemic area of *P. falciparum* and *P. Vivax*, both of which were taking chloroquine prophylaxis throughout their pregnancy until delivery. The seroprevalence of maternal PfVAR2CSA was 79.0% (38). The PfVAR2CSA significantly positively correlated between the mother and newborn antibody levels.

### Factors associated with the transfer of anti-malaria immunoglobulin

Additionally, gestational age, gravida status, and low birthweight were factors associated with either high or low transfer Examining factors that contributed to the transfer efficiency of antibodies against malaria antigen provides data or adds to existing knowledge regarding the maternal characteristic that impacts antibody transfer. Twenty studies reported on the factors related to mother, newborn antibodies, and transplacental transfer (12, 13, 15, 17, 19, 31–34, 36, 40, 47, 48, 51–56, 63). The specific factors associated with transfer efficiency, as well as those of IgG and its subclasses, are presented in **Table 2**.

**Table 2:** Factors associated with transplacental transfer of antibodies against malaria antigens.

Factor	Antigen	IgG class transfer				
		Total IgG	IgG1	IgG2	IgG3	IgG4
HIV infection	CSP	Reduced (33, 32)	Reduced (33)	–	–	–
	MSP	Reduced MSP2, MSP3 (33, 32) and MSP9 (56)	Reduced MSP1 (53)	–	Reduced in MSP1 (53)	–
	EBA	Reduced (56)	–	–	Reduced (53)	–
	AMA1	–	Reduced (53)	–	–	–
	Lysate	–	Reduced (53)	–	Reduced (53)	–
	CSP	Reduced (56)	–	–	–	–
Malaria	MSP	–	Reduced MSP1 (53)	–	Reduced MSP1 (53)	–
	EBA	Reduced (53)	Reduced (53)	–	–	–
	AMA	Reduced (53)	–	–	–	–
	Lysate	–	Reduced (53)	–	–	–
Placental malaria	MSP	Increased MSP1 & MSP2 (51)	–	Increased MSP2 (12)	–	–
	EBA	Increased (51)	–	–	–	–
	AMA1	Increased (51)	--	–	–	–
	DBL	Increased (51) (36)	–	–	–	–
	EXP1	Increased (31)	–	–	–	–
	Rh5		Increased			
Maternal hyper IgG	CSP	Reduced (33)	Reduced (33)	–	–	–
	MSP	Reduced MSP1 (33), & MSP2 (17, 33)	–	–	-Increased (40)	
	AMA1	Reduced (17, 33)	–	–	–	–
	GLURP-R2	Reduced (17, 33)	–	–	-Increased (40)	–

AMA-1, Apical Membrane Antigen 1, CSP, Circumsporozoite Protein DBLD, Duffy-binding like domains, EBA, Erythrocyte-binding antigen, EXP1, exported protein 1, MSP: merozoite surface proteins, RH4.2, Rh5, reticulocyte-binding protein homolog-5, GLURP RII: Glutamate Rich Protein R2.

Four studies reported that association of HIV infection and antibodies against malaria antigen in mothers, and newborns as well as their transfer efficiency (32, 33, 53, 56). HIV+ mothers had lower IgG antibodies against EBA181 and AMA1 (56), and IgG1 antibodies against CSP and MSP-1 (33) compared to HIV- mothers. HIV infection was also associated with reduced levels of MSP1 specific IgG antibodies in the newborn (33) and IgG1 levels against CSP, AMA-1, and MSP (33, 53), though IgG1antibodies against MSP-1 and EBA-175 were high (32). Maternal HIV reduced transfer of IgG antibodies against MSP2, MSP3, CSP (32, 33), MSP9, and EBA181 (56), and IgG1 antibodies against CSP (33), IgG1 to MSP1, lysate, AMA1, and IgG3 to MSP1, lysate, and EBA175 (53).

Malaria in pregnancy reduced maternal IgG antibodies against MSP2, EBA140, MSP3, MSPDBL1, and AMA1 (56), while malaria-negative mothers showed higher maternal IgG, IgG1, and IgG3 antibodies against QβMSP3, QβUB05, and QβUB05-MSP3 (48). neonates of uninfected mothers had higher QβUB05-specific antibodies (48). Transplacental transfer was reduced for IgG antibodies against CSP (56), EBA175, AMA1, IgG1 antibodies against EBA175, MSP1, lysate, and IgG3 antibodies against MSP1 (53).

Placental malaria was significantly elevates maternal IgG antibody against HSP40 (34) and IgG2 antibody against AMA1 (55) and newborn IgG antibody against (AMA1, EBA175, MSP1, and MSP2) (12) with subclass-specific increases (IgG1- Rh5, IgG2- MSP2, IgG3 - EBA175, and IgG4 - MSP142 (12) and RH22030 (55). Response varies by infection stage (acute, chronic, and past) (15). Materno-foetal transfer was high for IgG antibody against MSP1, EBA175, AMA1, DBLα (51), MSP2, EXP1 (31) and DBL 120 (36) but reduced in primigravida (52).

Maternal hypergammaglobulinemia was significantly correlated with increased newborn antibody levels (34), but reduced IgG antibody against EBA175, AMA1 (12, 53), lysate (53), CSP (33, 53), MSP1 (12, 33, 53), and MSP2 (12), and IgG1 and IgG3 antibody against EBA175, AMA1, lysate (53), and MSP1 (33). Transplacental transfer was reduced IgG antibody against CSP, MSP1 (33), AMA1, MSP2-FC27 and GLURP-R2 (17, 33) and IgG1 antibody against CSP (33), except high transfer for IgG3 antibody against (MSP-2, GLURP-R0/R20) in IgG3-H435 mothers (40).

of anti-malaria antibodies. Total IgG and antigen-specific antibodies increased in cord blood with gestational age (13, 19). Among primigravida, IgG1 antibody against PfAMA-1, PfMSP2, PfDBL5, and IgG3 to EBA175 RII and PfMSP2 were associated with reduced transfer (52). Low birth weight was associated with increased IgG2 antibodies against EXP1 and Rh4.2 in newborns. According to baby weight, a significant positive correlation was observed with IgG3 antibodies against QβUB05-MSP3 and IgG4 antibodies against QβMSP3 (48).

## Discussion

Although a little amount of IgG produced by the foetus (64), majority of circulating IgG antibodies in human newborns are of maternal origin and transferred across the placenta to provide early-life immunity until newborn IgG production takes over (65). Profiling and understanding factors associated with IgG transfer against malaria antigen is a priority. We conducted this review as no systematic review data were available on the transplacental transfer of antibodies against malarial antigens.

The majority of studies evaluated in this review were conducted in Sub-Saharan African countries (14, 15, 47, 48, 56, 60). We found that high IgG to CSP, AMA-1, MSP2 (17, 18) and its subclasses in mothers during delivery, while lower in the newborn. This reduction may be due to limited transfer efficiency or antigen-specific differences in IgG subclass distribution. IgG1 and IgG3 were dominant in mothers, but their transfer to the foetus was variable. IgG1 generally showed higher transfer than IgG3, and both were superior to IgG2 and IgG4. For instance, in newborns, antibodies against IgG1 and IgG3 to MSP1 and AMA1 were lower than mothers’ levels, which may influence neonatal protection (35, 42).

The efficiency of antibody transfer varied by antigen and infection status. For example, antibodies against CSP were transferred more than those against MSP3 (18). A systematic review conducted on transfer IgG to the foetus and preterm neonates compared shows that IgG1 was transferred more efficiently than IgG3, while IgG2 was transferred less efficiently (66). Wilson et al. also shows that malaria specific functional antibodies cross the placenta and then wane in infants overtime (67).

We identified that maternal HIV infection, malaria in pregnancy, placental malaria, hyperimmunoglobulin of maternal and birth weight were associated factors of transplacental transfer of antibodies against different malaria antigens. Maternal HIV infection was associated with reduced transplacental transfer of antibodies against malaria, in line with other studies (62). These may be attributed to HIV infection induces FcRn saturation (68), causing hypergammaglobulinemia, which impairs transplacental transfer of malaria IgG. Okoko et al. shows that maternal hypergammaglobulinemia was associated with a significant reduction in antibody transfer for herpes simplex virus 1, varicella-zoster virus, pneumococcus (69), and respiratory syncytial virus (70).

The effect of placental malaria on the transfer of antibodies against malaria antigen is variable. It reduced the transfer of antibodies against MSP1 and MSP3; however, it has been associated with a high level of transfer efficiency in the transfer of antibodies against MSP2 3D and MSP2 Fc27. Abnormalities in the placenta during placental malaria include structural damage such as thickening of the basement membrane, increased syncytial knotting, increased areas of fibrinoid necrosis, and dysregulated apoptosis of trophoblast, which can result placental insufficiency (71–73). The accumulation of *Plasmodium falciparum*-infected erythrocytes and immune cells in the intervillous spaces induces inflammatory responses (74, 75). These pathological changes may disrupt the integrity of the placental barrier and interfere with Fc receptor-mediated antibody transport mechanisms (74). The effect of placental malaria on transplacental transfer is antigen and strain-specific. For example, transfer of MSP2-Fc 27 IgG shows no significant difference between with and without placental malaria infection (17). In some cases, maternal infection increased total IgG levels (as with AMA1), but transfer to the foetus remained impaired. These may be due to variation in the relative abundance of the antigen in the circulating population, the strain used, the protein expression system used, and transmission intensities.

Hypergammaglobulinemia reduces transplacental transfer of anti-malaria antibodies. The possible reason may be that maternal IgG concentrations can saturate placental FcRn receptors, creating competitive inhibition that diminishes selective antibody transfer (65). This also observed in congenital human cytomegaly virus infection where total IgG transfer efficiency reduces to maternal hyperglobulinemia (76).

This systematic review has some limitation. First a quantitative meta-analysis of transplacental transfer ratios was not conducted due to heterogeneity of the outcome variable measurement and varied antigens. Second, although pooled prevalence estimates of malaria in mothers, placentas, and newborns were generated, it should be noted that this was not the primary objective of the review. The included studies were specifically selected based on their reporting of IgG antibody levels in mothers and newborns and their transfer efficiency and were not designed to serve as a representative population sample for malaria prevalence estimation. Consequently, the pooled prevalence findings should be interpreted with caution and not intended for broader generalizability beyond the context of the included study populations.

In conclusion, the efficiency of transplacental anti-malaria antibody transfer varied by antigen and IgG subclass. The risk factors associated with reduced transplacental transfer were found to be maternal HIV infection, peripheral malaria, placental malaria, hypergammaglobulinemia, low birth weight, and primigravida. Identifying the most efficiently transferred antigens and IgG subclasses could inform policies to prioritize those antigens during pregnant women’s vaccination against malaria antigens. These findings are critical for optimizing malaria vaccine strategies and enhancing protection for infants during early life.

Maternal prenatal vaccination is an emerging strategy that leverages transplacental antibody transfer to confer antigen-specific immunity to the foetus passively (77). Beyond characterizing the efficiency of malaria antibody transfer, future studies should incorporate system serology approaches to define the biophysical and functional properties of transferred antibodies, including their subclass distribution, Fc glycosylation patterns (78), and effector functions (e.g., complement activation, placental binding affinity).

## Data Availability

The original contributions presented in the study are included in the article/**Supplementary Material**. Further inquiries can be directed to the corresponding author.
